# Exposition aux Poussières et Santé Respiratoire des Broyeurs de Pierres dans la Province du Haut-Katanga en R. D. Congo

**DOI:** 10.48327/RTHG-RF19

**Published:** 2021-05-18

**Authors:** L.-K. Ngombe, R.-N. Nlandu, S.-N. Kazadi, B.-K. Ilunga, S.-W. Okitotsho, J.-B. K. Sakatolo, O.-L. Numbi, B. Danuser

**Affiliations:** 1 Département de recherche, Institut supérieur des techniques médicales de Lubumbashi, République démocratique du Congo; 2 Département de santé publique, Faculté de médecine, Unité de toxicologie, Université de Kamina, République démocratique du Congo; 3 Department of Public Health, Kagawa University School of Medicine, Miki, Japan; 4 Département de pédiatrie, Université de Lubumbashi, République démocratique du Congo; 5 Service of Occupational Medicine, Institute for Work and Health, University of Lausanne and Geneva, Epalinges-Lausanne, Switzerland; 6 École de santé publique, Université de Lubumbashi, Lubumbashi, République démocratique du Congo

**Keywords:** Symptômes respiratoires, Broyeurs de pierres, Exposition, Poussières de pierres, Carrières, Hernika, Wanchine, Lubumbashi, Haut-Katanga, République démocratique du Congo, Afrique sub-saharienne, Respiratory health, Stone crushers, Exposition, Stone dust, Stone-Pit, Hernika, Wanchine, Lubumbashi, Haut Katanga, Democratic Republic of Congo, Sub-Saharan Africa

## Abstract

Le but de ce travail était de déterminer la prévalence des symptômes et pathologies respiratoires, d'évaluer la fonction respiratoire, ainsi que d'élaborer des mesures préventives chez les broyeurs de pierres dans la province du Haut-Katanga, en République démocratique du Congo (RDC). Une étude transversale à visée analytique a été réalisée dans deux carrières. Au total, 293 broyeurs de pierres et 295 agents administratifs des bureaux communaux (sujets contrôles) ont participé à cette étude. Un questionnaire standardisé a été utilisé pour recueillir les données. La fonction respiratoire a été explorée à l'aide d'un débitmètre de pointe. Les particules fines (PM 2,5) et les composés organiques volatils (COV) dans les différents milieux de travail ont été également mesurés. La prévalence des symptômes respiratoires rapportée chez les broyeurs de pierres a été supérieure à celle du groupe contrôle. Une concentration élevée de PM 2,5 [197,5 μg/m^3^(185-210 μg/m^3^)] et des COV [1,95 mg/m^3^(1,5-2,4 mg/m^3^)] a été notée chez les broyeurs de pierres par rapport au groupe contrôle [respectivement 33,5 μg/m^3^(22-45 μg/m^3^); 0,75 mg/m^3^(0,6-0,9 mg/m^3^)]. Le débit expiratoire de pointe (DEP en l/mn) a été significativement réduit chez les broyeurs de pierres (421,84±88,18 l/mn) par rapport aux contrôles (450,37±70,90 l/min) (p<0,05). Il est donc impératif d'améliorer l'environnement et les conditions de travail des broyeurs des pierres.

## Abstract (en)

The aim of this study was to determine the prevalence of symptoms and respiratory diseases and to assess respiratory function in stones crushers stones in the province of Haut Katanga, Democratic Republic of Congo (DRC). This cross-sectional analytical study included 293 stones crushers and 295 office workers from public administrative services. A standardized respiratory health questionnaire was used, and lung function test performed with the use of peak flow-meters. PM 2.5 and volatile organic compounds (VOC) in different places of work have also been measured. The prevalence of respiratory symptoms was markedly higher in dust-exposed cement carriers than in controls. Air dust measurements showed higher mean PM 2.5 [197.5 μg/m^3^(range: 185-210 μg/m^3^) vs 33.5 μg/m^3^(range: 22-45 μg/m^3^)] and VOC [1.95 mg/m^3^(1.5-2.4 mg/m^3^) vs 0.75 mg/m^3^(0.6-0.9 mg/m^3^)] levels at stones crushers' worksites as compared with the control sites. On the other hand, peak flow rate (PEFR) was significantly lower in the exposed group as compared with the control group (421.84± 88.18 l/mn vs 450.37 ± 70.90 l/mn; p< 0.05). It is necessary to improve the environment and occupational conditions of stones crushers.

## Introduction

Les travailleurs des carrières sont susceptibles de développer des pathologies résultant de l'inhalation des poussières liées à leur activité ce qui entraîne de graves problèmes de santé dans nos pays peu nantis et sans protections de sécurité au travail. L'exposition professionnelle aux poussières est un phénomène bien connu surtout dans les pays en développement [1, 8]. Les émissions de poussières sont grandes dans les carrières de pierres et cela constitue une énorme source de pollution de l'air [12]. Cette exposition aux poussières de silice continue à se pratiquer malencontreusement surtout dans certains secteurs tels que les cimenteries, les mines, les carrières, les chantiers du bâtiment et des travaux publics (BTP) [8, 15]

**Fig. 1:**
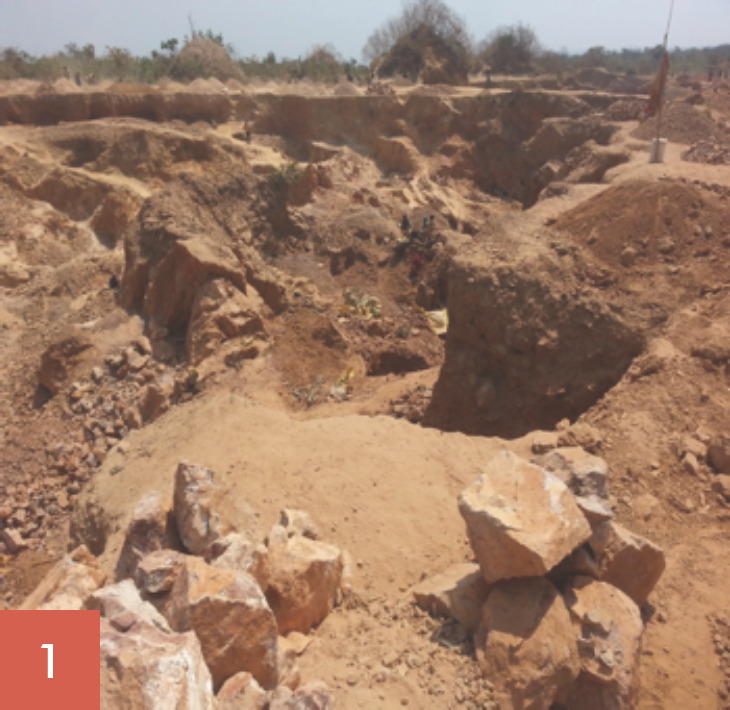
Vue d'ensemble de la carrière et Poste 1, concasseurs des roches en moellons Career overview and Station 1: stump crushing

**Fig. 2:**
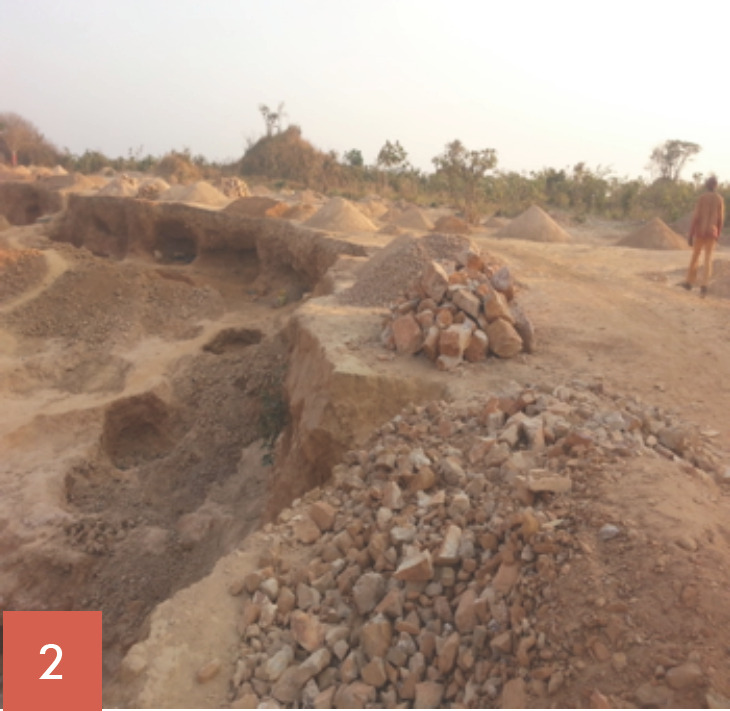
Vue d'ensemble de la carrière et Poste 1, concasseurs des roches en moellons Career overview and Station 1: stump crushing

**Fig. 3:**
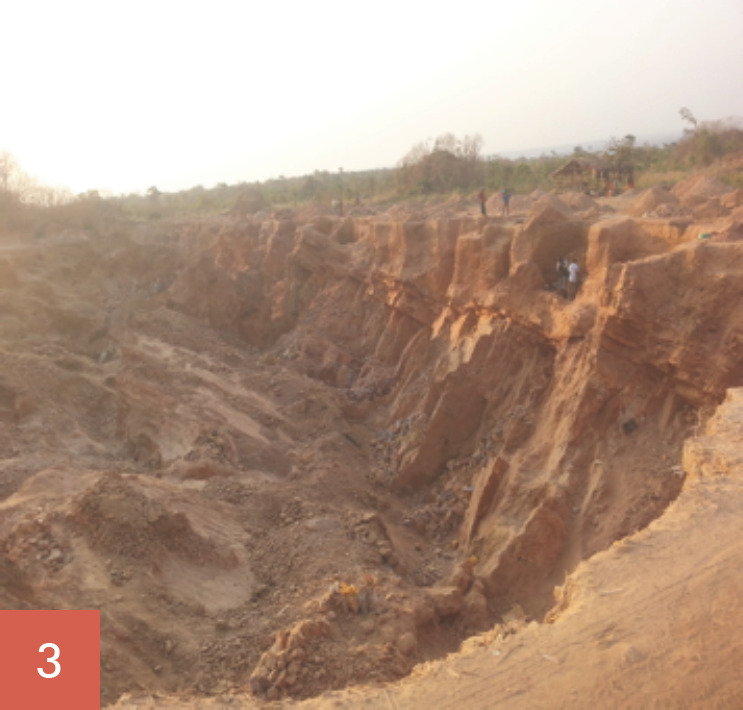
Vue d'ensemble de la carrière et Poste 1, concasseurs des roches en moellons Career overview and Station 1: stump crushing

**Fig. 4:**
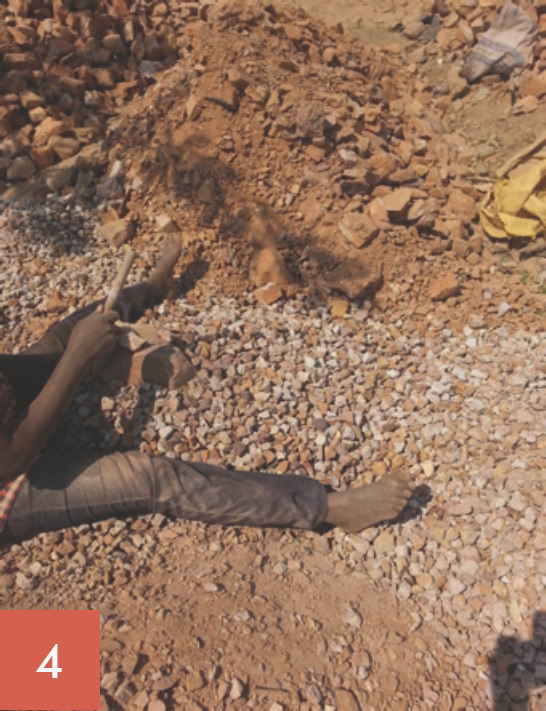
Concasseur des moellons en graviers Gravel crushing

**Fig. 5:**
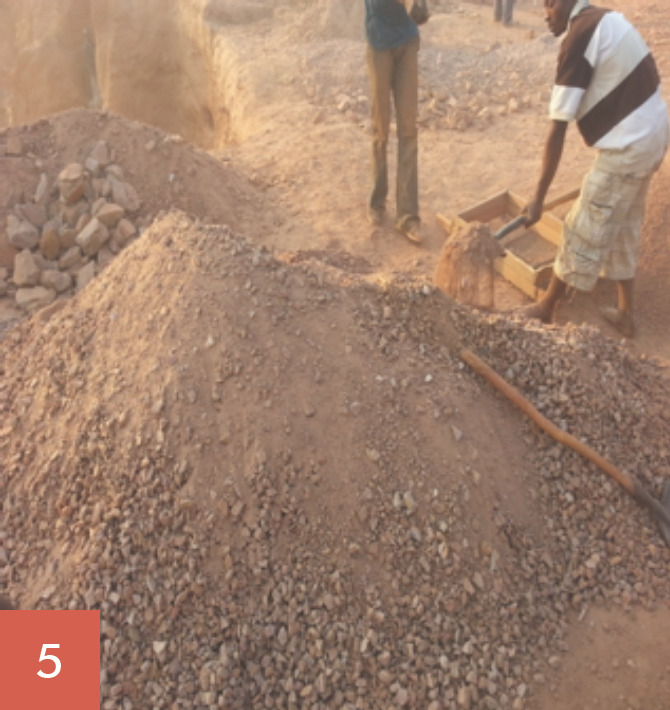
Poste 3, concasseurs des moellons, des graviers et production du sable Station 3, stumps and gravel crushing, and sand production

Les broyeurs de pierres oeuvrent tous les jours ouvrables de la semaine soit 5,5 jours/semaine. Ils ont des horaires de travail allant de 7 à 18 heures, soit 60 à 70 heures de travail/semaine. Leur tâche consiste à fracasser les grosses pierres (roches), puis à les concasser en moellons, en graviers et en sables (Fig. 1, 2, 3, 4, 5). Les broyeurs de pierres utilisent des matériels rudimentaires (marteaux, barres de fer, burins, tamis, bêches, etc.) pour transformer les roches en produits finis utilisables pour la construction. Ce processus de concassage libère de grandes quantités de poussières et de silice. Lors du chargement des matériaux dans les camions par ces mêmes broyeurs de pierre, de grandes quantités de poussières sont soulevées. Le sol de la carrière est recouvert de poussières de silice facilement remises en suspension par un simple courant d'air.

Les concasseurs ont trois postes de travail: concasseur des roches en moellons (poste 1: Fig. 1, 2, 3), concasseur des moellons en graviers (poste 2, Fig. 4) et concasseur des moellons, des graviers et production du sable (Fig. 3, Fig. 5).

Ils se reposent quand ils ont fini de charger les véhicules de leurs clients ou quand ils se sentent fatigués: ce sont des pauses de 20 à 60 minutes qui peuvent être prises plusieurs fois par jour ou en fonction du besoin. Ils travaillent sans masques, gants, système de pulvérisation d'eau et donc sans application des méthodes de prévention tant individuelles que collectives.

Les broyeurs de pierres sont des travailleurs indépendants qui s'auto-rémunèrent en fonction de leur production, avec une moyenne journalière allant de 5-10 dollars comme revenu quotidien.

Ils travaillent dans un environnement qui n'est régulé par aucune norme de santé au travail.

Il n'existe pas d'études épidémiologiques concernant les broyeurs de pierres congolais dont l'activité fait partie du secteur informel dans notre milieu, aussi le présent travail a pour objectif: d'investiguer la prévalence des symptômes et pathologies respiratoires ainsi que la fonction respiratoire chez les broyeurs de pierres, en comparaison avec les agents des bureaux du secteur de l'administration publique; de déterminer les facteurs de risque associés au broyage de pierres ainsi que les mesures de santé préventives à mettre en place chez ces travailleurs.

## Matériel et Méthodes

### Type, site et population d'étude

Il s'agit d'une étude transversale à visée analytique menée dans la ville de Lubumbashi qui est situé dans la province du Haut–Katanga. L'étude a été menée en 2016 sur une période de 2 mois, dans les carrières de Hernika et de Wanchine, allant du 1^er^ juillet au 31 août 2016.

Le secteur artisanal congolais de l'exploitation des pierres relève de l'économie informelle et n'a pas une réglementation conforme au code du travail congolais ni de service de médecine du travail. C'est pourquoi les enquêtés ont été recrutées sur leurs lieux de travail respectifs.

La population d'étude était composée de broyeurs de pierres et d'agents des bureaux de l'administration publique de la ville de Lubumbashi (groupe contrôle). La participation à une autre étude, le refus de remplir le formulaire du consentement éclairé et l'ancienneté inférieure à une année, au moment de cette étude étaient des critères d'exclusion de notre enquête.

### Questionnaire, examen médical et débitmètre de pointe

Les symptômes et pathologies respiratoires ainsi que les données socio-anthropométriques ont été recueillis à l'aide d'un questionnaire standardisé. Il s'agissait d'un questionnaire relatif aux symptômes bronchiques de l'Union internationale de lutte contre la tuberculose et les maladies respiratoires (1984) [6] et d'un questionnaire sur la rhinite allergique d'Annesi-Maesano [5]. L'enregistrement de la pression artérielle au moyen d'un appareil électronique (OMRON Hem8402) était effectué au bras gauche, soutenu à hauteur du coeur, le sujet s'étant reposé pendant au moins 10 minutes en position assise. La taille a été mesurée avec une toise et le poids au moyen d'une balance calibrée et vérifiée. Une auscultation anormale a été défini par la présence de râles (sibilants, crépitants et sous-crépitants etc.).

Une tente portable avait été installée dans les carrières comme dans les bureaux et toutes ces investigations ont été menées sur place. Le statut « fumeur » a été attribué à tout sujet ayant consommé du tabac durant plus d'une année, quel que soit le nombre de cigarettes par jour.

Les enquêteurs (deux médecins generalists et un infirmier) ont bénéficié d'une formation au préalable et l'enquête a été réalisée par entretien direct entre les enquêteurs et les personnes incluses dans l'étude.

Le débit expiratoire de pointe de chaque sujet a été mesuré à l'aide d'un appareil portable, le Wright Mini-Peak Flow Meter (Airmed; Clement Clarke International, Londres), tous les jours pairs de la semaine couplé aux questionnaires de l'enquête. Après un temps de repos, le sujet devait prendre une profonde inspiration et expiration forcées dans l'instrument, le nez étant fermé à l'aide d'un pince-nez. Chaque sujet a fait trois essais successifs et le meilleur des trois essais a été retenu comme meilleur DEP. Les particules PM 2,5 et les composés organiques volatils (COV) sur les sites de travail et dans les bureaux (pour le groupe contrôle) ont été évaluées à l'aide de l'appareil Bramc Air quality monitor BR-AIR-329 (Shandong, China). Trois mesures instantanées ont été effectuées dans les différents sites avec un intervalle de 30 minutes.

### Analyse statistique

Le logiciel SPSS 21.0 (SPSS Inc., Chicago, IL, USA) a permis l'encodage des données et les analyses ont été effectuées à l'aide de ce dernier. Les variables continues ont été présentées sous forme de moyennes et le test « t » de Student a été utilisé pour comparer les groupes d'étude. Les variables qualitatives et les valeurs, en revanche, ont été présentées sous forme de proportions, et le test du Chi carré a été utilisé pour comparer les proportions observées au sein des groupes. En outre, pour déterminer l'association entre les caractéristiques des broyeurs et les manifestations respiratoires rapportées, une analyse multivariée avec régression logistique a été privilégiée. Le seuil de signification a été fixé à p< 0,05.

## Résultats

### Mesures atmosphériques des particules fines

La mesure de particules fines a révélé des concentrations moyennes de PM 2,5 de l'ordre de 197,5 μg/m^3^ (extrêmes: 185-210 μg/m^3^) dans les carrières de pierres vs 33,5 μg/m^3^ (extrêmes: 22-45 μg/m^3^) dans le milieu de travail de contrôle, tandis que celles des COV étaient de 1,95 mg/m^3^ (extrêmes 1,5-2,4 mg/m^3^) dans les carrières de pierres vs 0,75 mg/m^3^ (limite: 0,6-0,9 mg/m^3^) dans le milieu de travail de contrôle, respectivement (p<0,05).

### Populations étudiées

Les travailleurs (broyeurs de pierres et agents administratifs du secteur public inclus) ayant plus d'une année d'ancienneté, travaillant tous les jours pairs de la semaine, ont été recrutés et retenus dans notre enquête sur la base des listes envoyées par les chefs des deux carrières. Chaque travailleur a reçu une fiche d'enquête anonyme sur laquelle apparaissait son numéro d'enregistrement dans la liste. Cette même fiche a servi à consigner les données recueillies à l'examen clinique.

Cent douze travailleurs ont été exclus (53 exposés et 59 contrôles), parmi lesquels 42 sujets ayant une ancienneté de moins d'une année, 33 sujets étant suivis dans une autre étude et 37 sujets ayant refusé de signer le formulaire de consentement éclairé.

Notre échantillon était composé de 588 travailleurs, dont 293 broyeurs de pierres et 295 agents de bureau. Sur les 6 grandes carrières des pierres qui ont été identifié dans la Province du Haut-Katanga, seuls deux étaient en activité durant notre étude.

Les broyeurs de pierres et les sujets contrôles avaient le même âge (34,13±11,65 et 33,89±9,31 ans), les mêmes indices de masse corporelle (22,57±2,92 et 23,95±3,53 kg /taille^2^) et la même pression artérielle (118,41 ±16,44 et 118,59 ±16,73; 72,85±12,69 et 70,97±11,24). Une différence significative (p<0,05) a été notée en ce qui concerne l'ancienneté, la durée du travail, la consommation d'alcool et de tabac. Concernant les paramètres cliniques, 41,3% des broyeurs de pierres contre 1% des témoins avaient une auscultation pulmonaire anormale, la différence étant très significative (p<0,001). Une proportion élevée (91,8%) des broyeurs de pierres n'avaient pas de certificats, ni de diplômes d'études. Par ailleurs, le débit expiratoire de pointe (DEP) moyen des broyeurs de pierres était très significativement inférieur à celui des contrôles (421,84±88,18 l/min contre 450,37±70,90 l/min; p<0,001) (Tableau 1).

**Tableau I:** Caractéristiques anthropométriques, cliniques et sociodémographiques DEP: débit expiratoire de pointe; DS: déviation standard; IMC: indice de masse corporelle; l/s: litre par seconde; PAD: pression artérielle diastolique; PAS: pression artérielle systolique; les valeurs de p indiquent le niveau de signification (0,05) Anthropometric, clinical and socio-demographic characteristics DEP: peak expiratory flow; DS: standard deviation; IMC: body mass index; L / s: liter per second; PAD: diastolic blood pressure; PAS: systolic blood pressure; the p values indicate the level of significance (0.05)

	**(N = 293)**	**(N = 295)**	
**Paramètres antdropométriques, cliniques et ceux liés au travail**	**Moyenne +/- DS**	**Moyenne +/- DS**	
âge (ans)	34,13 ± 11,65	33,89 ± 9,31	0,79
ancienneté (ans)	4,06 ± 5,49	7,58 ± 7,29	< 0,001
durée du travail (heure)	12,02 ± 0,13	9,82 ± 1,81	< 0,001
IMC (kg/taille^2^)	22,57 ± 2,92	23,95 ± 3,53	0,151
PAS	118,41 ± 16,44	118,59 ± 16,73	0,79
PAD	72,85 ± 12,69	70,97 ± 11,24	0,06
Paramètre du débitmètre de pointe			
DEP (l/minutes)	421,84 ±88,18	450,37±70,90	<0,001
paramètres sociodémographiques			
Niveau d'études			
primaire-secondaire incomplet	269 (91,8 %)	201 (68,1 %)	
diplômé	24 (8,2 %)	94 (31,9 %)	<0,001
alcool (Oui)	152 (51,9 %)	41 (13,9 %)	< 0,001
tabac (Oui)	115 (39,2 %)	24 (8,1 %)	< 0,001
auscultation pulmonaire (pathologique)	121 (41,3 %)	3 (1,0 %)	< 0,001

Les broyeurs de pierres présentaient une prévalence de symptômes respiratoires plus élevée par rapport au groupe contrôle avec une différence statistiquement significative: sifflement (22,9% contre 7,9%), essoufflement au repos (37,9% contre 2%), essoufflement après effort (38,2 contre 2%), toux le matin (44% contre 2%), bronchite chronique (23,2% contre 1,7%), rhinite (71,7% contre 15,3%). Par contre, la prévalence de l'asthme (3,1% contre 2,1%) était plus élevée chez les sujets exposés mais sans une différence significative (p<0,05) (Tableau II).

**Tableau II:** Prévalence des symptômes et pathologies respiratoires RP: ratio de prévalence; IC: intervalle de confiance; les valeurs de p indiquent le niveau de signification (0,05). Prevalence of respiratory symptoms and pathologies RP: prevalence ratio; IC: confidence interval; the p values indicate the level of significance (0.05)

Symptômes et pathologies respiratoires	Broyeurs de pierres	Non-exposés	RP	X^2^	IC [95 %]	P
	**(N = 293)**	**(N = 295)**				
sifflement	67 (22,9 %)	19 (7,8 %)	1,64	25,75	[1,4-1,91]	<0,001
essoufflement/repos	111 (37,9 %)	6 (2,0 %)	2,45	118,53	[2,17-2,77]	<0,001
essoufflement/après effort	112 (38,2 %)	6 (2,0 %)	2,46	120,03	[2,18-2,78]	<0,001
asthme	9 (3,1 %)	7 (2,4 %)	1,13	0,27	[0,73-1,76]	0,39
toux/matin	129 (44,0 %)	6 (2,0 %)	2,64	146,55	[2,32-2,99]	<0,001
crachat/matin	145 (49,5 %)	8 (2,7 %)	2,78	167,08	[2,43-3,19]	<0,001
bronchite chronique	68 (23,2 %)	5 (1,7 %)	2,13	62,57	[1,89-2,39]	<0,001
rhinite	210 (71,7 %)	45 (15,3 %)	3,30	190,51	[2,72-4,01]	<0,001

Concernant les différents postes de travail, le poste 2 (concasseur des moellons en graviers) enregistrait une prévalence élevée d'essoufflements au repos par rapport au poste 1 (concasseur des roches en moellons) et 3 (concasseur des moellons, des graviers et production de sable) avec une différence statistiquement significative. La prévalence de la toux le matin et la bronchite chronique était élevée dans le poste de travail 3 par rapport aux postes 2 et 1 (p<0,05) (Tableau III).

**Tableau III:** Association entre les postes de travail des broyeurs des pierres et les symptômes respiratoires Poste 1: concassage des moellons; Poste 2: concassage des graviers; Poste 3: concassage des moellons, des graviers et production de sable; les valeurs de p indiquent le niveau de signification (0,05. ORa: odds ratio ajusté (95%); DEP Δa: différence ajustée; pa: p ajustée. Comparison between the characteristics of stone crushers and respiratory symptoms Station 1: stump crushing; Station 2: gravel crushing; Station 3: stumps and gravel crushing, and sand production; the p values indicate the level of significance (0.05). ORa: adjusted odds ratio (95%): confidence interval, p <0.05; DEP Δa: adjusted difference; pa: p adjusted.

**Symptômes et pathologies respiratoires**	**Broyeurs de pierres**			
	Poste 1 (N = 78)	Poste 2 (N = 77)	Poste 3 (N = 138)	
	N (%) ORa [95%	N (%) ORa [95%]	N (%) ORa [95%]	p pa
sifflement	11 (14,1%) 1,04 [0,46-2,38]	19 (24,7 3,07 [1,5-6,3]	37 (26.8%) 2,47 [1,32-4,6]	0,09 0,0018
essoufflement/repos	20 (25,6%) 12,04 [4,52-32,04]	33 (42,9%) 29,7 [11,64 -75,8]	58 (42,0%) 26,78 [10,98-65,32]	0,03 < 0,001
essoufflement/après effort	22 (28,2%) 15,06 [5,73-39,62]	31 (40,3%) 27,33[10,70-69,81]	59 (42,8%) 29,71[12,19-72,39]	0,09 < 0,001
toux le matin	23 (29,5%) 16,82 [6,44-43,99]	36 (46,8%) 39,08 [15,36-99,39]	70 (50,7%) 42,18 [17,37-102,42]	0,009< 0,001
crachat le matin	31 (39,7%) 19,04 [8,09-44,77]	40 (51,9%) 32,91 [14,19-76,34]	74 (53,6%) 34,59 [15,67-76,36]	0,13 < 0,001
bronchite chronique	13 (16,7%) 9,03 [3,03-26,87]	12 (15,6%) 9,35 [3,14-27,86]	43 (31,2%) 20,87 [7,87-55,34]	0,01 < 0,001
rhinite	54 (69,2%) 13,42 [7,33-24,58]	59 (76,6%) 18,30 [9,75-34,34]	97 (70,3%) 14,16 [8,44-23,75]	0,52 < 0,001
DEP Δa	61,27 [39,89-82,65]	39,92 [18,83-61,01]	67,69 [50,07-85,31]	< 0,001

Après ajustement pour certains paramètres (âge et tabac), l'analyse multi variée a révélé que tous les postes de travail des broyeurs de pierres avaient par rapport au groupe contrôle, un risque élevé de symptômes et de pathologies pulmonaires (p<0,05). En outre, chaque poste de travail chez les broyeurs de pierres avait une influence significative sur la fonction respiratoire de ces derniers avec p<0,05.

## Discussion

Cette étude a montré que la prévalence des symptômes et des pathologies respiratoires était remarquablement élevée chez les broyeurs de pierres par rapport au groupe contrôle. Le processus de transformation des roches en matériaux de construction (moellons, graviers, sables) génère de grandes quantités de poussières néfastes pour la santé.

Cette étude révèle des concentrations élevées de PM 2,5 et de COV dans l'environnement de travail des carrières de pierre. Les valeurs instantanées de PM 2,5 dépassent de loin les valeurs recommandées par l'Organisation mondiale de la santé (OMS) (25 μg/m^3^ moyenne sur 24 heures) [19], et par le Congrès national américain d'hygiénistes industriels (ACGIH) (0,05 mg/m^3^ moyenne journalière) [4].

Nous avons constaté que les travailleurs n'utilisaient pas les équipements de protections requis (masques‚ gants‚ lunettes), souvent par faute de moyens, par manque d'éducation ou par manque d'information ou de la non disponibilité de ces derniers. Tous ces facteurs contribuent à la prévalence élevée des symptômes et pathologies respiratoires. Nos observations sont soutenues par la littérature [3, 9, 11] qui souligne le manque d'éducation, un niveau socio-économique bas et la non-disponibilité des équipements de protection dans le secteur informel, notamment chez les ouvriers des carrières.

Nos résultats cliniques sont supérieurs à ceux trouvés par Isara et al et Asgedom et al [2, 10], respectivement au Nigeria et en Ethiopie. Notre étude montre une prévalence de bronchite chronique de 23,2% et de 71,7% de rhinite. Ces résultats sont similaires à ceux trouvés chez les mineurs du coltan [11]. Par ailleurs, nos résultats sont également comparables à ceux trouvés par d'autres auteurs chez différents groupes de travailleurs exposés aux poussières [4, 16].

En outre, une prévalence élevée de consommation d'alcool (51,9%) et de tabac (39,2%) chez les broyeurs de pierres par rapport au groupe contrôle (13‚9% et 8‚1%) a été notée. Ce phénomène est fréquent chez les travailleurs de force exerçant leurs activités dans un contexte de précarité des conditions de travail. Ces travailleurs se livrent à une forte consommation d'alcool et de tabac afin d'oublier leurs mauvaises conditions de travail. Ce constat a également été fait au cours des études menées au Katanga [7] et à Kinshasa [17].

Concernant le débitmètre de pointe, les résultats de notre enquête ont montré qu'il y avait une différence significative entre le DEP des broyeurs de pierres et les contrôles. Des études antérieures ont également trouvé des résultats similaires [11, 16]. Une association a été trouvée entre la profession des broyeurs de pierres (y compris les différents postes de travail), les manifestations respiratoires et la diminution du DEP. Selon la littérature, les COV jouent un rôle dans la détérioration des voies respiratoires, tandis que les fortes concentrations des particules fines sont responsables d'altérations de l'état de santé des sujets exposés aux poussières environnementales [13, 14, 18]. D'où l'importance de réduire les concentrations des poussières dans l'environnement de travail des broyeurs de pierres, d'améliorer les conditions de travail, les mesures hygiéniques et de définir nos valeurs limites d'exposition aux poussières et aux composés organiques volatils dans notre pays (RD Congo).

Il est très important d'utiliser des systèmes de pulvérisation d'eau dans les carrières afin de réduire les concentrations élevées de poussières [13]. Nous recommandons l'application de cette pratique dans les mesures de lutte contre les poussières dans notre pays, surtout dans le secteur informel artisanal où la situation sanitaire des artisans demeure une catastrophe.

Cette enquête fournit aux chercheurs et aux décideurs des politiques sanitaires de nos pays, des informations pertinentes au sujet de l'environnement de travail, de la prédominance des manifestations respiratoires et de l'absence des mesures préventives chez les sujets exposés aux poussières des carrières de pierre.

Ce type d'étude transversale ne peut pas démontrer le lien causal entre l'exposition aux poussières, l'environnement et les manifestations respiratoires chez les travailleurs. De plus, nos résultats se focalisent seulement sur l'atteinte bronchique et les tests utilisés n'ont pas permis de différencier asthme (réversible) et BPCO (TVO non réversible). Ceci constitue une faiblesse et limite de notre étude. À cela, s'ajoute l'effet du travailleur sain qui peut conduire à sous-estimer les prévalences des symptômes cliniques et l'importance du risque professionnel en général. Mais un sur-recrutement possible de personnes se sachant atteintes de maladie respiratoire, et espérant des soins et remèdes doit aussi être considéré comme un biais. Une autre faiblesse de notre étude est le fait de ne pas savoir si les travailleurs étaient à poste fixe ou tournaient.

Il est également important de savoir que les pneumopathies infiltrantes diffuses sont probablement fréquentes dans cette population exposée à la silice et responsables d'une partie des dyspnées. Compte tenu d'un plateau technique limité, ces dernières n'ont pas été recherchées dans notre étude. Des études longitudinales seront nécessaires pour bien investiguer la santé des travailleurs des carrières et mines exposés aux poussières et à la silice.

## Conclusion

Cette étude confirme la prévalence élevée des affections respiratoires des broyeurs de pierres des carrières (Hernika et Wanchine) due aux mauvaises conditions de travail, au manque d'équipements de protection individuels ainsi que de systèmes de pulvérisation d'eau contre les poussières en suspension dans l'air.

Il s'avère important d'adopter une médecine de travail adaptée, une prévention technique rigoureuse et d'appliquer la législation dans ce secteur afin de réguler l'exposition des travailleurs selon les normes.

## Conflits D'intérêts

Les auteurs ne déclarent aucun conflit d'intérêts.

## References

[1] Aigbedion I, Iyayi SE (2007). Environmental effect of mineral exploitation in Nigeria. Int J Phys Sci.

[2] Asgedom AA, Bråtveit M, Moen BE (2019). High Prevalence of Respiratory Symptoms among Particleboard Workers in Ethiopia: A Cross-Sectional Study. Int J Environ Res Public Health.

[3] Aliyu AA, Shehu AU (2006). Occupational hazards and safety measures among stone quarry workers in northern Nigeria. Nig Med Pract.

[4] American Conference of Governmental Industrial Hygiene. (2009). Threshold limits values for chemical substances and physical agents in the work environment and biological exposure indices. Cincinnati Conference.

[5] Annesi-Maesano I, Didier A, Klossek M, Chanal I, Moreau D, Bousquet J (2002). The score for allergic rhinitis (SFAR): a simple and valid assessment method in population studies. Allergy.

[6] Burney PG, Laitinen LA, Perdrizet S, Huckauf H, Tattersfield AE, Chinn S, Poisson N, Heeren A, Britton JR, Jones T (1989). Validity and repeatability of the IUATLD (1984) Bronchial Symptoms Questionnaire: an international comparison. Eur Respir J.

[7] Elenge MM, De Brouwer C (2011). Identification of hazards in the workplaces of Artisanal mining in Katanga. Int J Occup Med Environ Health.

[8] Fatusi A, Erhabor G (1996). Occupational health status of sawmill workers in Nigeria. J R Soc Health.

[9] Hentschel T, Hruschka F, Priester M (2002). Global report on artisanal and small scale mining. Mining, minerals and sustainable development. World Business Council for Sustainable Development.

[10] Isara AR, Adam VY, Aigbokhaode AQ, Alenoghena IO (2016). Respiratory symptoms and ventilatory functions among quarry workers in Edo state, Nigeria. Pan Afr Med J.

[11] Ngombe LK, Ngatu NR, Kakoma SJ, Nyembo C, Mbelambela EP, Moribe RJ, Wembonyama S, Danuser B, Oscar-Luboya N (2018). Respiratory health of dust-exposed Congolese coltan miners. Int Arch Occup Environ Health.

[12] Olusegun O, Adeniyi A, Adeola GT (2009). Impact of Granite Quarrying on the Health of Workers and Nearby Residents in Abeokuta Ogun State, Nigeria. Ethiop J Environ Stud & Manage.

[13] Gottesfeld P, Tirima S, Anka SM, Fotso A, Nota MM (2019). Reducing Lead and Silica Dust Exposures in Small-Scale Mining in Northern Nigeria. Ann Work Expo Health.

[14] Sivacoumar R, Jayabalou R, Subrahmanyam YV, Jothikumar N, Swarnalatha S (2001). Air pollution in stone crushing industry, and associated health effects. Indian J Environ Health.

[15] Toloba Y, Sissoko BF, Badoum G, Nenzeko RT, Ouattara K, Soumaré D, Sidibé S, Diallo S (2014). Poumon de puisatier au Mali pendant la décennie 2001-2010. Rev Pneumol Clin.

[16] Tyagi R, Chandarana P (2013). Peak expiratory flow rate value: construction labourers. Int J Sci Res.

[17] Wangata J, Elenge M, De Brouwer C (2014). Les accidents du travail dans le transport urbain en commun de la ville province de Kinshasa, République Démocratique du Congo: une étude transversale descriptive. Pan Afr Med J.

[18] Wong JY, De Vivo I, Lin X, Christiani DC (2014). Cumulative PM(2.5) exposure and telomere length in workers exposed to welding fumes. J Toxicol Environ Health A.

[19] World Health Organisation (WHO). Ambient (outdoor) air quality and health 2016. http://www.who.int/mediacentre/factsheets/fs313/en/.

